# Validation of CTS5 model in large-scale breast cancer population and the impact of menopausal and HER2 status on its prognostic value

**DOI:** 10.1038/s41598-020-61648-1

**Published:** 2020-03-13

**Authors:** Changjun Wang, Chang Chen, Yan Lin, Yidong Zhou, Feng Mao, Hanjiang Zhu, Xiaohui Zhang, Songjie Shen, Xin Huang, Xuefei Wang, Bin Zhao, Jing Yang, Qiang Sun

**Affiliations:** 1 Department of Breast Surgery, Peking Union Medical College Hospital, Chinese Academy of Medical Sciences and Peking Union Medical College, Beijing, 100730 China; 2 Department of Dermatology, University of California, San Francisco, CA 94143–0989 United States; 3 Department of Emergency Service, Peking Union Medical College Hospital, Chinese Academy of Medical Sciences and Peking Union Medical College, Beijing, 100730 China

**Keywords:** Breast cancer, Cancer

## Abstract

Clinical Treatment Score post-5 years (CTS5) is a promising prognostic tool to evaluate late recurrence risk for breast cancer. Our study aimed to validate its prognostic value in large-scale population and explore the impact of menopausal and HER2 status on CTS5 model. We performed a retrospective cohort study using the Surveillance, Epidemiology, and End Results (SEER) database. Survival analyses were conducted to assess the prognostic value of CTS5 in different breast cancer subgroups in terms of overall survival (OS) and breast cancer specific survival (BCSS) after five years. A total of 23,168 breast cancer patients with positive hormone receptor (HoR) were enrolled. Postmenopausal and premenopausal patients were 13,686 and 9,482, respectively. Taking CTS5 score as a continuous variable, it had significant positive correlation with poor prognosis beyond five years in both postmenopausal and premenopausal subgroups. Nevertheless, for HER2+ postmenopausal patients, the model has less effective prognostic value on long-term BCSS [HR1.177 (95%CI 0.960–1.443), *p* = 0.117]. Using CTS5 score as a categorical variable, HER2- patients with high-risk level revealed significant poor survival in terms of both BCSS and OS, irrespective of menopausal status. Our study showed the CTS5 model could be a useful prognostic tool for predict long-term survival in HoR+/HER2- patients. And further large-scale studies are warranted to assess its prognostic value for HER2+ patients and develop novel prediction model for late recurrence risk estimation.

## Introduction

Breast cancer had the highest incidence among all the female malignancies worldwide^1,2^. The introduction of screening mammography and the advent of novel therapeutic agents largely prolonged the survival of breast cancer patients, especially for hormone receptors (HoR) positive tumors. Patients with HoR+ tumors currently received five years adjuvant endocrine therapy as standard treatment. However, more than 50% of patients relapsed or metastasized after the completion of standard endocrine therapy. Recently, several studies proposed that prolonged endocrine therapy beyond five years could improve the prognosis^3–7^. But extended endocrine therapy may also raise safety concerns as increasing unacceptable adverse effects and the debate on over-treatment. Given that common clinicopathological markers could not provide enough power for long-term recurrence prediction, it was crucial to find a novel modality for long-term recurrence risk evaluation and clinical decision making^8^. Additionally, the existing data on personalized strategy for HoR+ patients to extend endocrine therapy beyond five years were still contradictory, and the optimal duration and protocol remained unclear. Study by Dowsett *et al*.^9^ developed a concise prognostic tool to estimate risk of late distant recurrence (Clinical Treatment Score post-5 years [CTS5]) on the basis of clinicopathologic parameters. The authors used data from the ATAC (Arimidex, Tamoxifen, Alone or in Combination) trial^10^ as the training set and the BIG (Breast International Group) 1–98 trial as the validation set^11^. The final CTS5 prediction model was CTS5 = 0.438 * nodes + 0.988 *(0.093 *size − 0.001 * size^2^+ 0.375* grade + 0.017* age). The model divided 5 to 10 years of long-term recurrence risk into three groups based on calculation results: low risk, <5%; medium risk, 5% to 10%; and high risk, >10%. However, the CTS5 model was developed based on retrospective data, its calibration and discrimination needed further external evaluation in large-scale breast cancer datasets. Given that the CTS5 model was build based on post-menopausal and HER2- patients, its prognostic value for the other subgroups remained undetermined. Therefore, the present study intended to use large-scale data from SEER database to assess the power of CTS5 as a prediction model and the impact of menopausal and HER2 status on its performance.

## Results

### Demographics and clinicopathological characteristics of study population

The final analysis included 23,168 patients, including 9,482 premenopausal, and 13,686 postmenopausal women, respectively (“postmenopausal” was defined as age ≥ 55). Postmenopausal subgroup had less lymph node involvement, smaller tumor size and lower histological grade than premenopausal patients (Post- vs. Pre-: lymph node negative74.7% vs. 64.2%, *p* < 0.001; tumor less than 10 mm 27.8% vs. 20.3%, *p* < 0.001; tumor grade I 29.8% vs. 23.8%, *p* < 0.001). HER2+ were more common in premenopausal patients (Post- vs. Pre-: 11.0% vs. 15.4%; *p* < 0.001), and more premenopausal patients received chemotherapy (Post- vs. Pre-: 29.9% vs. 55.6%; *p* < 0.001). Besides, patients were prone to receive less radiotherapy in premenopausal group (Post- vs. Pre-: 61.0% vs. 52.8%, *p* < 0.001). Deaths due to non-breast causes were more common in postmenopausal patients (Post- vs. Pre-: 1.0% vs. 0.1%; *p* < 0.001), and the proportion of deaths due to breast cancer was comparable in two groups (Post- vs. Pre-: 0.5% vs. 0.7%; *p* = 0.094). Details for demographics and clinicopathological characteristics of study population were listed in Table 1. If the cutoff to define post-menopausal subgroup was set to 60 years old, the above demographic and clinicopathological characteristics exhibited a similar pattern as cutoff was set to 55 years old. Data were shown in Supplementary Table S1.

**Table 1:** Demographic and Clinical Characteristics of included Patients (“postmenopausal” was defined as age ≥ 55).

Characteristics	Postmenopausal No. (%) (n = 13686)	Premenopausal No. (%) (n = 9482)	*p*
**Age**	55–80	18–54	—
Median	64	48	
**Nodal status (No. of positive nodes)**			<0.001
Negative	10225 (74.7)	6089 (64.2)	
1	1682 (12.3)	1453 (15.3)	
2–3	970 (7.1)	1055 (11.1)	
4–9	589 (4.3)	673 (7.1)	
> 9	220 (1.6)	212 (2.2)	
**Grade**			<0.001
Well (I)	4077 (29.8)	2255 (23.8)	
Intermediate (II)	6654 (48.6)	4604 (48.6)	
Poor (III)	2955 (21.6)	2623 (27.7)	
**Tumor size (mm)**			<0.001
< 10	3810 (27.8)	1924 (20.3)	
10 < =T < 20	5830 (42.6)	3538 (37.3)	
20 < =T < 30	2364 (17.3)	2071 (21.8)	
> =30	1682 (12.3)	1949 (20.6)	
**HER2**			<0.001
+	1508 (11.0)	1462 (15.4)	
−	12178 (89.0)	8020 (84.6)	
**Chemotherapy**	4095 (29.9)	5273 (55.6)	<0.001
**Radiotherapy**	8346 (61.0)	5003 (52.8)	<0.001
**Death due to breast cancer**	75 (0.5)	65 (0.7)	0.094
**Death due to other reason**	135 (1.0)	12 (0.1)	<0.001

### Late recurrence risk distribution of study population

Patients could be divided into three subgroups based on CTS5 risk score: low risk (<5%), intermediate risk (5–10%), and high risk (>10%). The distribution of risk categories in SEER cohort was shown in Table 2 (“postmenopausal” was defined as age ≥ 55). For premenopausal patients, it was noteworthy that all small tumors (<10 mm) were at low-risk group (97.4%). And the majority of node-negative patients (76.9%) were also classified as low-risk recurrent group, while 25.6% patients who had only one lymph node-positive was classified as a high-risk group, and 31.8% of 2–3 lymph node-positive patients were classified as intermediate and low-risk. For tumor grade, only 38.3% of patients with grade III were at high-risk for recurrence. For postmenopausal patients, 71.2% of node-negative patients were classified as low-risk, and almost all patients with ≥4 positive lymph nodes had high risk of recurrence. And 59.1% of patients with T2 tumors and 45.5% of patients with grade III tumor were classified as high-risk. Supplementary Table S2 summarized the risk distribution when Age ≥ 60 years was used to define postmenopausal status.

**Table 2:** Distribution of risk categories in SEER cohort According to Tumor Size, Grade, and Nodal Involvement (“postmenopausal” was defined as age ≥ 55).

Characteristic	No. (%)			Total No.
	**Low risk**	**Intermediate risk**	**High risk**	
**Premenopausal**				
Total	5235 (55.2)	2185 (23.0)	2062 (21.7)	9482
**Size, mm**				
<10	1874 (97.4)	27 (1.4)	23 (1.2)	1924
10–20	2895 (73.1)	721 (18.2)	347 (8.7)	3963
>20	466 (13.0)	1437 (40.0)	1692 (47.0)	3595
**Grade**				
Well	1899 (84.2)	213 (9.4)	143 (6.3)	2255
Intermediate	2642 (57.4)	1047 (22.7)	915 (19.9)	4604
Poor	694 (26.5)	925 (35.3)	1004 (38.3)	2623
**Nodal involvement**				
0	4682 (76.9)	1265 (20.8)	142 (2.3)	6089
1	462 (31.8)	646 (44.5)	345 (23.7)	1453
2–3	75 (7.1)	262 (24.8)	718 (68.1)	1055
4–9	14 (2.1)	12 (1.8)	647 (96.1)	673
>9	2 (1.0)	0 (0)	210 (99.1)	212
**Postmenopausal**				
**Total**	7698 (56.2)	3298 (24.1)	2690 (19.7)	13686
**Size, mm**				
<10	3708 (97.3)	65 (1.7)	37 (1.0)	3810
10–20	3827 (60.5)	1943 (30.7)	552 (8.7)	6322
>20	163 (4.6)	1290 (36.3)	2101 (59.1)	3554
**Grade**				
Well	3515 (86.2)	397 (9.7)	165 (4.0)	4077
Intermediate	3648 (54.8)	1825 (27.4)	1181 (17.7)	6654
Poor	535 (18.1)	1076 (36.4)	1344 (45.5)	2955
**Nodal involvement**				
0	7285 (71.2)	2374 (23.2)	566 (5.5)	10225
1	371 (22.1)	730 (43.4)	581 (34.5)	1682
2–3	30 (3.1)	193 (10.0)	747 (77.0)	970
4–9	10 (1.7)	0 (0)	579 (98.3)	589
>9	2 (1.0)	1 (0.5)	217 (98.6)	220

### Survival analysis for different subgroups

**Figure 1:**
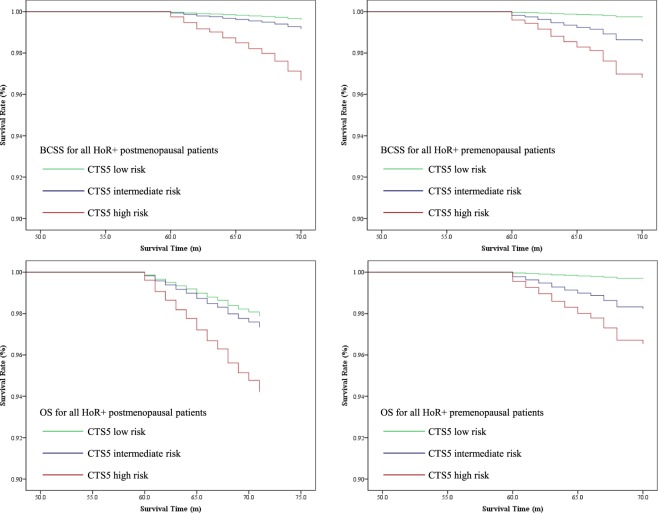
Survival curves of OS and BCSS according to CTS5 risk category for both premenopausal and postmenopausal patients with HoR+.

**Figure 2:**
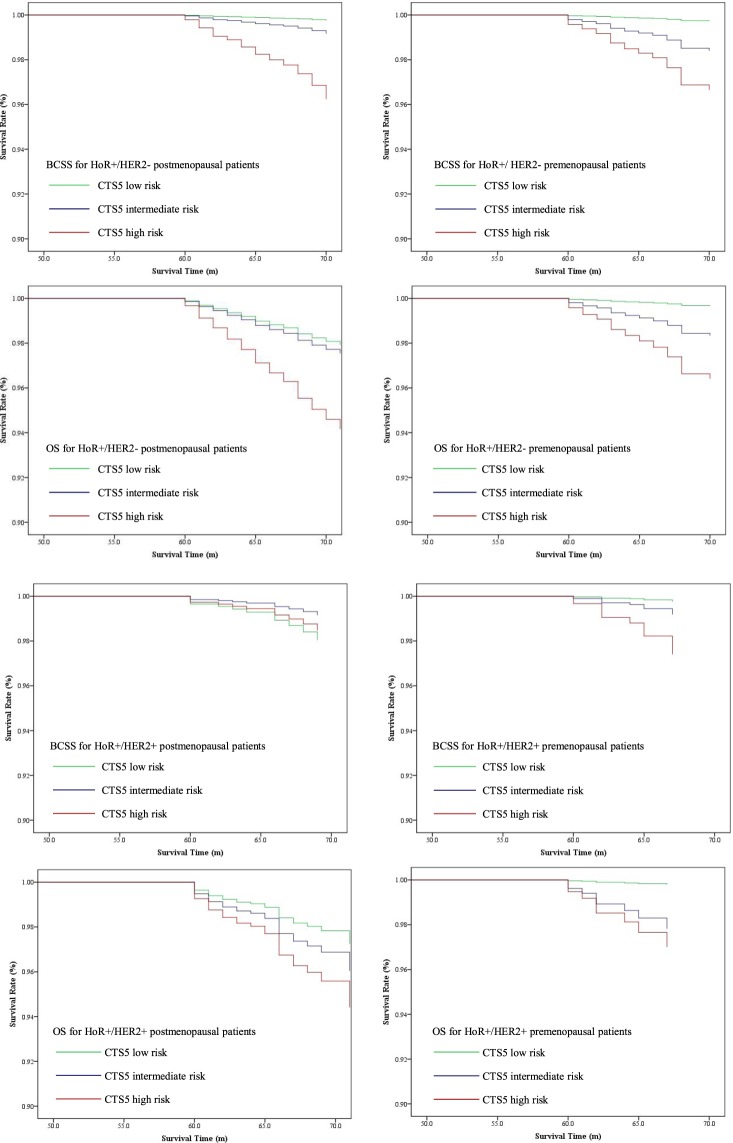
Survival curves of OS and BCSS according to CTS5 risk category for different breast cancer intrinsic subtypes.

All the patients included were followed up more than 60 months (median 65 m). The overall survival (OS) rate and breast cancer specific survival (BCSS) rate were 99.2% and 99.3%, respectively. The survival curves with different CTS5 risk score were shown in Figs. 1 and 2 (“postmenopausal” was defined as age ≥ 55) and Supplementary Figs. S1 and S2 (“postmenopausal” was defined as age ≥ 60).

The Cox analysis taking CTS5 as a continuous variable showed the risk score had a positive correlation with poor prognosis in postmenopausal patients [HR1.186 (95% CI 1.126–1.250), *p* < 0.001 for OS and HR1.277 (95% CI 1.203–1.356), *p* < 0.001 for BCSS] (Table 3). However, in HER2+ subgroup, the CTS5 model did not have a strong discriminating power for long-term recurrence risk [HR1.176 (95% CI 1.032–1.339), *p* = 0.015 for OS and HR1.177 (95% CI 0.960–1.443), *p* = 0.117 for BCSS]. For premenopausal patients, the CTS5 score was negatively corelated with long-term prognosis, irrespective of HER2+ status and chemotherapy administration. Details were summarized in Table 3.

**Table 3:** Survival analyses for BCSS and OS in different subgroups of HoR+ patients (CTS5 as continuous variable, “postmenopausal” was defined as age ≥ 55).

CTS5 as continuous variable	Postmenopausal				Premenopausal			
	OS		BCSS		OS		BCSS	
	HR (95% CI)	*p*	HR (95% CI)	*p*	HR (95% CI)	*p*	HR (95% CI)	*p*
All patients	1.186 (1.126–1.250)	<0.001	1.277 (1.203–1.356)	<0.001	1.289 (1.227–1.354)	<0.001	1.297 (1.231–1.366)	<0.001
HER2+	1.176 (1.032–1.339)	0.015	1.177 (0.960–1.443)	0.117	1.384 (1.217–1.574)	<0.001	1.443 (1.244–1.673)	<0.001
HER2-	1.185 (1.118–1.255)	<0.001	1.287 (1.211–1.369)	<0.001	1.277 (1.208–1.350)	<0.001	1.281 (1.209–1.357)	<0.001
Chemotherapy	1.101 (0.999–1.214)	0.053	1.135 (1.016–1.268)	0.025	1.252 (1.182–1.326)	<0.001	1.260 (1.186–1.338)	<0.001
No chemotherapy	1.401 (1.308–1.502)	<0.001	1.545 (1.409–1.694)	<0.001	1.405 (1.202–1.643)	<0.001	1.391 (1.159–1.671)	<0.001

HR: hazard ratio; BCSS: breast cancer specific survival; OS: overall survival; HoR+: hormone receptor-positive; HER2+: HER2-positive; HER2−: HER2-negetive.

Taking CTS5 as a categorical variable, it also showed good discriminating power for HER2- patients. (Table 4). It is noteworthy that for postmenopausal patients with HER2+, CTS5 cannot clearly distinguish low-to-high-risk groups in terms of both OS and BCSS [HR 2.060 (95% CI 0.864–4.910), *p* = 0.258 for OS; HR 0.777 (95% CI 0.219–2.753), *p* = 0.583 for BCSS]. For premenopausal patients with HER2+, the CTS5 score also had strong prognostic value [HR 0.777 (95% CI 0.219–2.753), *p* = 0.043 for OS; HR 11.096 (95% 1.365–90.195), *p* = 0.044 for BCSS). Details were summarized in Table 4.

**Table 4:** Survival analyses for BCSS and OS in different subgroups of HoR+patients (CTS5 as categorical variable, “postmenopausal” was defined as age ≥ 55).

A. BCSS					
		Low risk	Intermediate risk HR (95% CI)	High risk HR (95% CI)	*p*
Postmenopausal	All HoR+ patients	Reference	2.185 (1.055–4.526)	8.815 (4.922–15.788)	<0.001
	HER2+ patients	Reference	0.429 (0.087–2.126)	0.777 (0.219–2.753)	0.583
	HER2− patients	Reference	3.339 (1.407–7.924)	15.204 (7.401–31.233)	<0.001
Premenopausal	All HoR+ patients	Reference	5.539 (2.409–12.740)	12.382 (5.787–26.495)	<0.001
	HER2+ patients	Reference	3.454 (0.313–38.092)	11.096 (1.365–90.195)	0.044
	HER2− patients	Reference	6.002 (2.469–14.589)	12.724 (5.616–28.826)	<0.001
**B. OS**					
		**Low risk**	**Intermediate risk HR (95% CI)**	**High risk HR (95% CI)**	***p***
Postmenopausal	All HoR+ patients	Reference	1.254 (0.873–1.801)	2.769 (2.041–3.758)	<0.001
	HER2+ patients	Reference	1.447 (0.558–3.751)	2.060 (0.864–4.910)	0.258
	HER2− patients	Reference	1.187 (0.798–1.764)	2.865 (2.063–3.978)	<0.001
Premenopausal	All HoR+ patients	Reference	5.653 (2.690–11.876)	11.169 (5.621–22.876)	<0.001
	HER2+ patients	Reference	10.207 (1.229–84.790)	14.080 (1.784–111.142)	0.043
	HER2− patients	Reference	4.958 (2.210–11.122)	10.822 (5.202–22.513)	<0.001

HR: hazard ratio; BCSS: breast cancer specific survival; OS: overall survival; HoR+: hormone receptor-positive; HER2+: HER2-positive; HER2−: HER2-negetive.

All the results above were using 55 years old to define menopausal status. And for setting 60 years as cutoff, CTS5 was also proved to a powerful prognostic tool (Supplementary Tables S3 and S4).

## Discussion

Prolonging endocrine therapy may reduce the occurrence of recurrence and metastases beyond five years for HoR+ patients, but potentially increased risk of endometrial complications (including cancer), thromboembolic events, fractures, cardiovascular disease and other adverse effects^12–15^. CTS5 model was a promising prediction tool for assessing long-term recurrence risk, which could help us select appropriate patients for extending endocrine therapy. However, its prognostic value had not been verified in large-scale populations or subgroups. We carried out this study to validate the model by 23,168 patients from SEER database, and evaluated the impact of HER2 status and menopausal status on its prediction performance.

The present study validated that CTS5 had a good discriminating power for long-term recurrence risk of HER2- patients, irrespective of menopausal status. This was concordant with the study by Richman *et al*.^16^ that CTS5 could be used for both pre- and post-menopausal patients. However, the HER2 status has a great impact on its performance. The high-risk patients generally had large tumors, poor tumor differentiation and lymph node metastasis. Parameters like age, tumor size, tumor grade, and lymph node were involved in the CTS5 model, among which lymph node involvement played an important role. In this study, almost all patients with lymph node metastasis ≥ 4 were classified as high-risk. So, the lymph node metastasis is a strong predictor of late recurrence, which is consistent with previous studies^17,18^. Compared with the original model population, the present study also included premenopausal patients. By Cox univariate analysis, it was proved that the model can distinguish the low, middle and high risk of long-term recurrence well in premenopausal patients with HoR+/HER2−, even better than in postmenopausal patients. So menopausal status did not significantly affect the model’s performance, indicating its application area could be extended to premenopausal women. For patients who received chemotherapy, our data showed the CTS5 model did not have a strong discriminating power for postmenopausal women in terms of OS. It could probably be attributed to the absence of other independent prognostic indicators in the original model, such as KI67^19,20^. Thus, novel model that integrated immunohistochemical features and gene signature (such as IHC4 model) would be helpful to determine the late recurrence risk and deliver personalized medicine^21^.

Menopausal status had a great impact on clinical decision making, especially for HoR+ patients that needed endocrine therapy. However, the definition of menopause remained controversial. Generally, age ≥ 55 was considered to be post-menopausal. According to this standard, the present study proved that CTS5 model was effective for both pre- and post-menopausal HoR+/HER2− patients. To further validate the above results, another cutoff using age ≥ 60 according to NCCN guideline was adopted^22^. This criterion had higher specificity for diagnosis of menopause than the previous one, indicating the postmenopausal subgroup was more homogeneous and could provide more reliable conclusion. Using either age ≥ 55 or 60 as cutoff, CTS5 was proved to have good performance to evaluate late recurrence risk irrespective of menopausal status. Additionally, it was of note that menopause was a complex biological phenomenon and age as a single indicator may not be sufficient to clearly define menopausal status. It may inevitably introduce bias to the final conclusion and a prospective large-scale population study may be needed for further validation.

HER2 was generally considered as an important prognostic indicator. The present study also evaluated the impact of HER2 status on CTS5 model and there was no significant correlation between CTS5 score and survival of postmenopausal HER2+ patients. Although CTS5 as categorical variable had certain prognostic value in HER2+ premenopausal women, it should be cautious to apply this finding to clinical practice. HER2 itself is an independent prognostic risk factor, indicating a high degree of malignancy and worse prognosis^23–25^. It may mask the effects of the other parameters, especially for late recurrence, with the existence of plenty of confounding factors. Meanwhile, the advance of HER2 targeted therapy such as trastuzumab, even dual-target therapy has largely improved the prognosis of HER2+ patients. Thus, risk prediction models only included common parameters, such as age, tumor size, lymph node status and tumor grade, may not be sufficient to serve as an effective prediction model for HER2+ patients. Further studies may need more homogeneous patient cohort and more clinical meaningful parameters to develop powerful prediction models for HER2+ patients.

For determination of the optimal endocrine therapy duration, genetic testing may provide additional valuable prognostic information on late recurrence. For instance, Oncotype DX recurrence score, using reverse transcription PCR (RT-PCR) assays incorporates 21 genes (including six housekeeping genes) related to proliferation, survival, invasion and estrogen receptor signaling^26^. Although it was widely used for early recurrence evaluation, retrospective study using TransATAC data proved the performance of Oncotype DX alone for predicting late recurrence risk was not satisfactory^18,27,28^. Another useful genetic tool, PAM50, combines the expression levels of 50 genes and tumor size to define the breast cancer intrinsic subtype and provide proliferation information by proliferation-related genes^29^. Studies showed that PAM50 exhibited potential to predict both early and late recurrence risk up to 10-year recurrence ^28,30^. However, this result was limited to postmenopausal, HER2- patients. Sestak *et al*.^31^ demonstrated the prognostic value for PAM50 of late recurrence in a combined analysis of the ATAC and ABCSG 8 trial populations. The prognostic performance of PAM50 plus CTS5 was superior to CTS5 alone. Future studies should integrate prognostic information from multiple dimensions, such as demography, clinicopathological parameters, gene signature, epigenetics and so on, to develop comprehensive and precise prediction models.

Our study also has several limitations. First, due to the retrospective nature of the present study, selection bias could not be totally eliminated. The mismatch of baseline characteristics could not be totally justified with multivariate analyses. Secondly, the SEER database did not incorporate treatment information regarding adjuvant/neoadjuvant therapies and duration. Different treatment options served as an important confounding factor, especially for ovarian function suppression. Thirdly, the definition of menopause remained controversial and SEER registry did not include patient menstrual status, it may potentially introduce bias. And we used two cutoffs (55 and 60 years) to evaluate the impact of menstrual status on CTS5 performance, and the final conclusions of the two grouping methods were comparable. Finally, the median follow-up time was 65 months, it was not long enough to evaluate late recurrence and validate the benefit of extending endocrine treatment for 10 year. Since only patients after 2010 had HER2 records, this was one of the reasons why the present study only included patients between 2010 and 2013 to ensure at least five years follow-up. Future studies with longer observation period may provide more robust evidence for late recurrence.

In conclusion, our study proved the CTS5 model is a useful tool to evaluate late recurrence risk in HoR+/HER2-negetive patients, irrespective of menopausal status. Further large-scale studies are warranted to assess its prognostic value for HER2+ patients and develop novel prediction model for late recurrence risk estimation.

## Methods

### Study population

**Figure 3:**
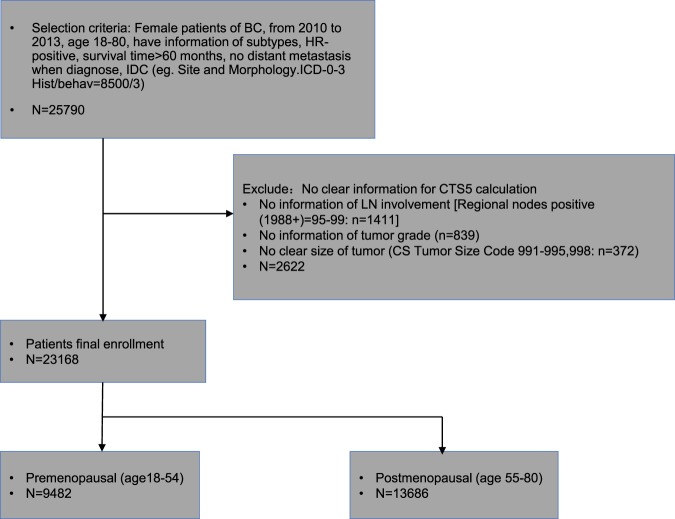
Enrollment of cohort BC: breast cancer; HoR: hormone receptor; IDC: invasive ductal carcinoma; LN: lymph node.

Female patients with invasive breast cancer in the SEER database from 2010–2013 who had no distant metastasis and had been followed up for ≥5 years were included. The SEER database includes morbidity and survival data routinely collected from multiple population-based cancer registries^28^. SEER* Stat version 8.3.5 was used to generate the data sheet including individual cancer records, patient characteristics, and the following variables: patient identification number, year of diagnosis, age, ethnicity, tumor size, nuclear grade, lymph node metastasis status, TNM Staging, estrogen receptor (ER) status, progesterone receptor (PR) status, HER2 status, radiotherapy, cause-specific death classification, other causes of death, survival months, marital status, radiotherapy and chemotherapy. Patient record without lymphatic involved data, tumor size or tumor grade were excluded (Fig. 3). Subgroup analyses were carried out based on menopausal status (menopausal was defined as age ≥ 55 years) and HER2 status.

### Outcome of interests

We calculated CTS5 score for each patient using the equation of CTS5 = 0.438 * nodes + 0.988 *(0.093 *size − 0.001 * size^2^ + 0.375* grade + 0.017* age) and then divided into three groups using the cutoff points for low-(CTS5 <3.13 for risk <5%), intermediate-(3.13 to 3.86 for risk of 5%–10%), and high-risk (CTS5 >3.86 for risk >10%)groups from this model^9^. The primary endpoint was breast cancer-specific death. SEER defines mortality data on the basis of the International Classification of Diseases Revisions 8–10^32–34^. The SEER cause of death recode was used to categorize the death as breast cancer specific death, other cancer death, death as a result of heart disease, or noncancer cause of death. The OS and BCSS were calculated as the time period from the date of diagnosis until the last date for which completed vital status data were available. The data regarding deaths were ascertained from death certificates that are coded by state health departments and/or state vital records for each SEER region^35^.

### Statistical analysis

The demographical and clinicopathological variables such as age, histological grade, tumor size, N-stage, chemotherapy and radiation therapy were assessed by t-test for continuous data and Pearson Chi-square test for categorical data. Kaplan-Meier method and Cox proportion hazard regression were used to perform survival analysis. BCSS was defined as the time between breast cancer diagnosis and death due to breast cancer, while OS was the period between diagnosis and death due to all causes (including breast cancer). Statistical analyses were performed using SPSS statistical software version 22 and R (3.6.0) software. All the statistical tests were two-sided, and statistical significance was defined as *p* value < 0.05.

## Supplementary information

Supplementary Information.

## Data Availability

The datasets generated during and/or analyzed during the current study are available from the corresponding author on reasonable request.
