# Supplementary material for: Validation of CTS5 model in large-scale breast cancer population and the impact of menopausal and HER2 status on its prognostic value
Source: Sci Rep. 2020 Mar 13;10:4660. doi: 10.1038/s41598-020-61648-1 (PMC7070010; doi:10.1038/s41598-020-61648-1)

Supplementary information

Validation of CTS5 model in large-scale breast cancer population and the impact of menopausal and HER2 status on its prognostic value

Changjun Wang[1+], Chang Chen[1+], Yan Lin[1+], Yidong Zhou[1], Feng Mao[1], Hanjiang Zhu[2], Xiaohui Zhang[1], Songjie Shen[1], Xin Huang[1], Xuefei Wang[1], Bin Zhao[1], Jing Yang [3], Qiang Sun[1*]

[1] Department of Breast Surgery, Peking Union Medical College Hospital, Chinese Academy of Medical Sciences and Peking Union Medical College, Beijing, 100730, China

[2]Department of Dermatology, University of California, San Francisco, CA 94143–0989, United States

[3]Department of Emergency Service, Peking Union Medical College Hospital, Beijing, 100730, China

[*]Corresponding author. Email: sunqiangpumch@sina.com

[+]These three authors contributed equally to this article

Supplementary Table S1. Demographic and Clinical Characteristics of included Patients ("postmenopausal" was defined as age ≥ 60)

**Characteristics**	**Postmenopausal No.(%)(n=10045)**	**Premenopausal No.(%)(n=13123)**	***p***
**Age**	60-80	18-59	-
**Median**	67	50	
**Nodal status (No. of positive nodes)**			<0.001
**Negative**	7676 (76.4)	8638(65.8)	
**1**	1181 (11.8)	1954(14.9)	
**2-3**	672(6.7)	1353(10.3)	
**4-9**	375 (3.7)	887(6.8)	
**>9**	141(1.4)	291(2.2)	
**Grade**			<0.001
**Well (I)**	3023 (30.1)	3309(25.2)	
**Intermediate (II)**	4945 (49.2)	6313(48.1)	
**Poor (III)**	2077 (20.7)	3501(26.7)	
**Tumor size (mm)**			<0.001
**<10**	2910 (30.0)	2824(21.5)	
**10<=T<20**	4352 (43.3)	5016(38.2)	
**20<=T<30**	1681 (16.7)	2754(21.0)	
**>=30**	1102 (11.0)	2529(19.3)	
**HER2**			<0.001
+	1062 (10.6)	1908(14.5)	
-	8983(89.4)	11215(85.5)	
**Chemotherapy**	2532 (25.2)	6836(52.1)	<0.001
**Radiotherapy**	6139 (61.1)	7210(54.9)	<0.001
**Dead of breast**	46 (0.5)	85(0.6)	0.056
**Dead of other reason**	130 (1.3)	26(0.2)	<0.001

Supplementary Table S2. Distribution of risk categories in SEER cohort According to Tumor Size, Grade, and Nodal Involvement (“postmenopausal” was defined as age ≥ 60)

Characteristic	No.(%)			Total No.
	Low risk	Intermediate risk	High risk	
**Premenopausal**				
**Total**	7299 (55.6)	3020 (23.0)	2804 (21.4)	13123
**Size, mm**				
**<10**	2748 (97.3)	42 (1.5)	34 (1.2)	2824
**10-20**	4010 (71.9)	1090 (19.5)	480 (8.6)	5580
**>20**	541 (11.5)	1888 (40.0)	2290 (48.5)	4719
**Grade**				
**Well**	2801 (84.6)	313 (9.5)	195 (5.9)	3309
**Intermediate**	3608 (57.1)	1488 (23.6)	1217 (19.3)	6313
**Poor**	890 (25.4)	1219 (34.8)	1392 (39.8)	3501
**Nodal involvement**				
**0**	6601 (76.4)	1804 (20.9)	233 (2.7)	8638
**1**	588 (30.1)	865 (44.3)	501 (25.6)	1954
**2-3**	87 (6.3)	338 (25.1)	928 (68.6)	1353
**4-9**	19 (2.1)	12 (1.4)	856 (96.5)	887
**>9**	4 (1.4)	1 (0.3)	286 (98.3)	291
**Postmenopausal**				
**Total**	5634 (56.1)	2463 (24.5)	1948 (19.4)	10045
**Size, mm**				
**<10**	2834 (97.4)	50 (1.7)	26 (0.9)	2910
**10-20**	2712 (57.6)	1574 (33.5)	419 (8.9)	4705
**>20**	88 (3.6)	839 (34.5)	1503 (61.9)	2430
**Grade**				
**Well**	2613 (86.4)	297 (9.8)	113 (3.7)	3023
**Intermediate**	2682 (54.2)	1384 (28.0)	879 (17.8)	4945
**Poor**	339 (16.3)	782 (37.7)	956 (46.0)	2077
**Nodal involvement**				
**0**	5366 (69.9)	1835 (23.9)	475 (6.2)	7676
**1**	245 (20.7)	511 (43.3)	425 (36.0)	1181
**2-3**	18 (2.7)	117 (17.4)	537 (79.9)	672
**4-9**	5 (1.3)	0 (0)	370 (98.7)	375
**>9**	0 (0)	0 (0)	141 (100)	141

Supplementary Table S3. Survival analyses for BCSS and OS in different subgroups of HoR+ patients (“postmenopausal” was defined as age ≥ 60; CTS5 as continuous variable)

	**Postmenopausal**				**Premenopausal**			
**CTS5**	OS		BCSS		OS		BCSS	
**as continuous variable**	HR（95%CI）	*p*	HR（95%CI）	*p*	HR（95%CI）	*p*	HR（95%CI）	*p*
**All patients**	1.171(1.103,1.244)	<0.001	1.282(1.193,1.377)	<0.001	1.282(1.226,1.341)	<0.001	1.288(1.225,1.354)	<0.001
**HER2+**	1.183(1.028,1.360)	0.019	1.189(0.947，1.493)	0.135	1.336(1.184,1.508)	<0.001	1.388(1.207,1.595)	<0.001
**HER2-**	1.166(1.089,1.248)	<0.001	1.291(1.198，1.392)	<0.001	1.275(1.214,1.340)	<0.001	1.276(1.208,1.349)	<0.001
**Chemotherapy**	1.088(0.970,1.221)	0.149	1.142(1.009，1.294)	0.036	1.235(1.167,1.306)	<0.001	1.232(1.156,1.313)	<0.001
**No chemotherapy**	1.135(1.249,1.464)	<0.001	1.507(1.331，1.706)	<0.001	1.478(1.329,1.642)	<0.001	1.511(1.345,1.697)	<0.001

HR: hazard ratio; BCSS: breast cancer specific survival; OS: overall survival; HoR+: hormone receptor-positive; HER2+: HER2-positive; HER2-: HER2-negetive.

Supplementary Table S4. Survival analyses for BCSS and OS in different subgroups of HoR+ patients (“postmenopausal” was defined as age ≥ 60; CTS5 as categorical variable)

A．BCSS

		Low risk	Intermediate risk HR（95%CI）	High risk HR（95%CI）	*p*
Postmenopausal	All HoR+ patients	Reference	3.900（1.623，9.37）	5.181（2.533，10.60）	<0.001
	HER2+ patients	Reference	<0.001（0.00，Inf）	0.279（0.564，12.89）	0.208
	HER2- patients	Reference	5.428（2.219，13.28）	5.705（2.563，12.70）	<0.001
Premenopausal	All HoR+ patients	Reference	3.863（2.057，7.254）	4.988（3.044，8.173）	<0.001
	HER2+ patients	Reference	5.154（1.289，20.61）	6.317（1.928，20.7）	0.0023
	HER2- patients	Reference	3.624（1.769，7.424）	4.764（2.753，8.244）	<0.001

B．OS

		Low risk	Intermediate risk HR（95%CI）	High risk HR（95%CI）	*p*
Postmenopausal	All HoR+ patients	Reference	2.207（1.316，3.702）	2.101（1.301，3.391）	0.0024
	HER2+ patients	Reference	2.692（0.892，8.117）	2.852（1.0361，7.85）	0.0425
	HER2- patients	Reference	2.058（1.129，3.721）	1.901（1.095，3.303）	0.0226
Premenopausal	All HoR+ patients	Reference	3.656（2.087，6.405）	4.921（3.196，7.576）	<0.001
	HER2+ patients	Reference	3.429（1.106，10.63）	4.408（1.735，11.20）	0.0018
	HER2- patients	Reference	3.607（1.886，6.899）	4.956（3.042，8.074）	<0.001

HR: hazard ratio; BCSS: breast cancer specific survival; OS: overall survival; HoR+: hormone receptor-positive; HER2+: HER2-positive; HER2-: HER2-negetive.

Supplement Figure S1. Survival curves of OS and BCSS according to CTS5 risk category for both premenopausaland postmenopausal patientswith HoR+(“postmenopausal” was defined as age ≥ 60)

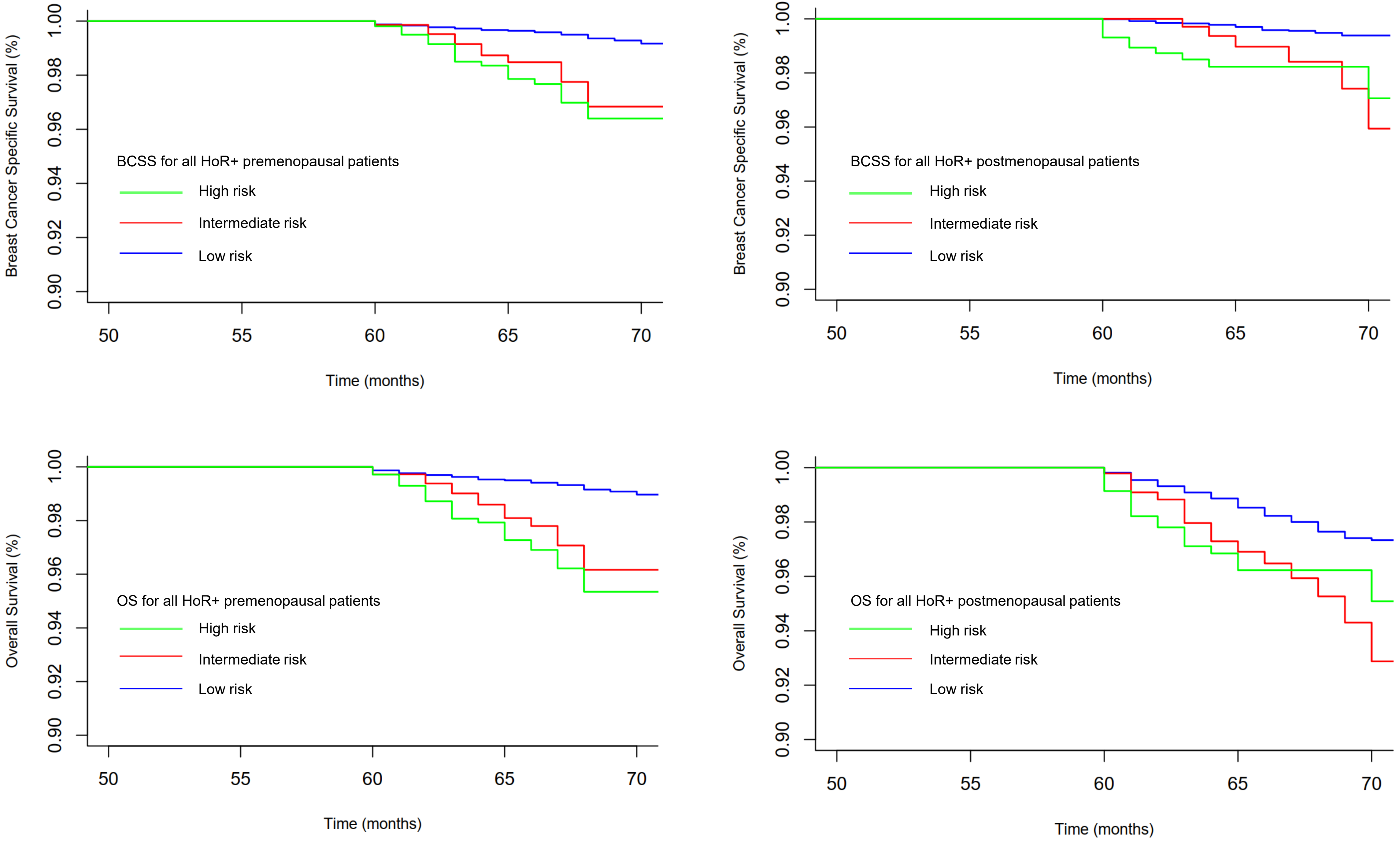

Supplement Figure S2. Survival curves of OS and BCSS according to CTS5 risk category for different breast cancer subtypes (“postmenopausal” was defined as age ≥ 60)

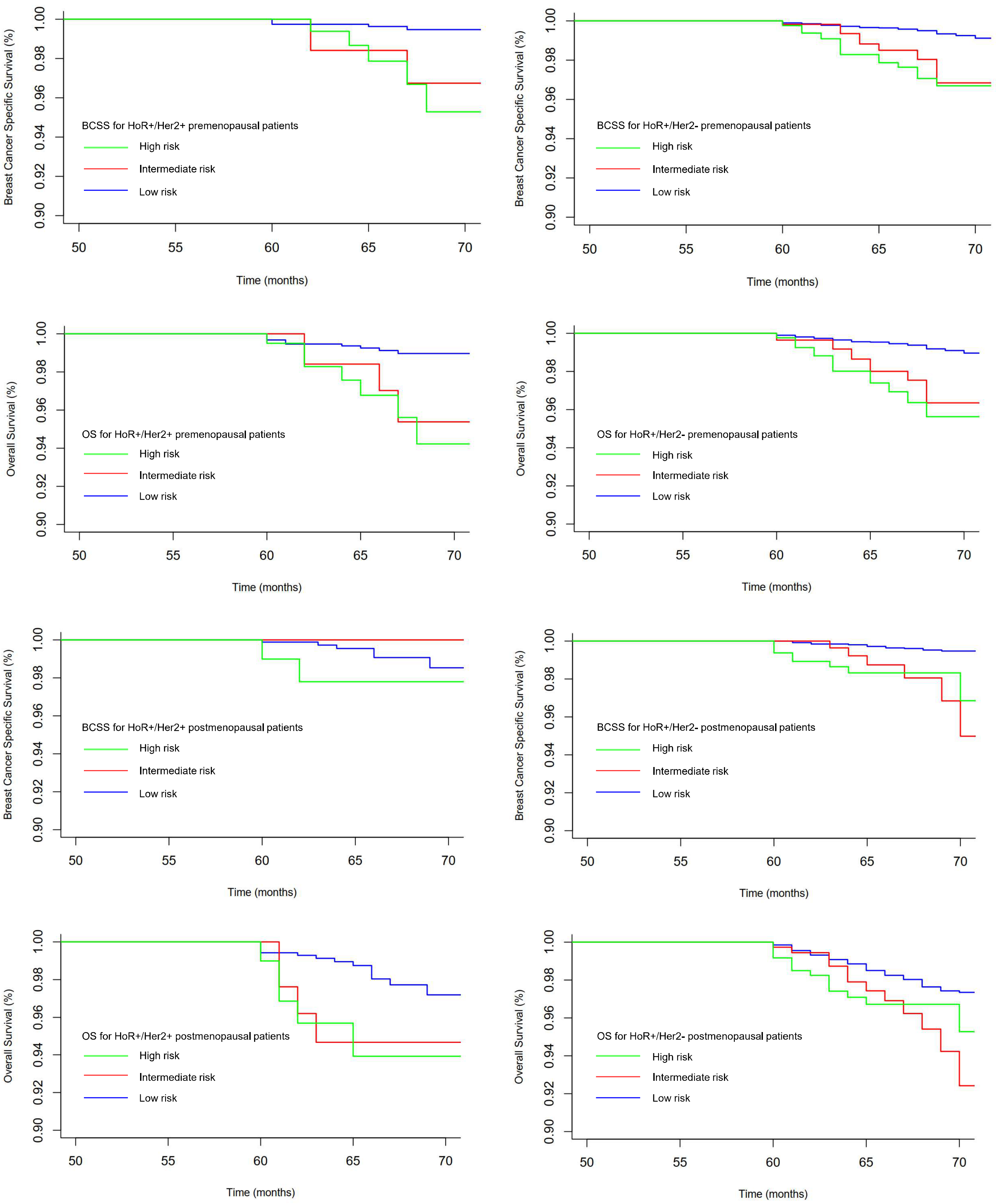

Supplement: Supplementary file 1 — Supplementary Information. [file 41598_2020_61648_MOESM1_ESM.pdf]
